# Safety of Acupuncture: Overview of Systematic Reviews

**DOI:** 10.1038/s41598-017-03272-0

**Published:** 2017-06-13

**Authors:** Malcolm W. C. Chan, Xin Yin Wu, Justin C. Y. Wu, Samuel Y. S. Wong, Vincent C. H. Chung

**Affiliations:** 1 Hong Kong Institute of Integrative Medicine, The Chinese University of Hong Kong, Hong Kong, China; 2 Faculty of Medicine, University of Toronto, Toronto, Canada; 3 Jockey Club School of Public Health and Primary Care, The Chinese University of Hong Kong, Hong Kong, China; 4 Hong Kong Branch of the Chinese Cochrane Centre, The Chinese University of Hong Kong, Hong Kong, China

## Abstract

Acupuncture is increasingly used worldwide. It is becoming more accepted by both patients and healthcare providers. However, the current understanding of its adverse events (AEs) is fragmented. We conducted this overview to collect all systematic reviews (SRs) on the AEs of acupuncture and related therapies. MEDLINE and EMBASE were searched from inception to December 2015. Methodological quality of included reviews was assessed with a validated instrument. Evidence was narratively reported. Seventeen SRs covering various types of acupuncture were included. Methodological quality of the reviews was overall mediocre. Four major categories of AEs were identified, which are organ or tissue injuries (13 reviews, median: 36 cases, median deaths: 4), infections (11 reviews, median: 17 cases, median deaths: 0.5), local AEs or reactions (12 reviews, median: 8.5 cases, no deaths were reported), and other complications such as dizziness or syncope (11 reviews, median: 21 cases, no deaths were reported). Minor and serious AEs can occur during the use of acupuncture and related modalities, contrary to the common impression that acupuncture is harmless. Serious AEs are rare, but need significant attention as mortality can be associated with them. Referrals should consider acupuncturists’ training credibility, and patient safety should be a core part of acupuncture education.

## Introduction

In China, it is estimated that nine hundred million traditional Chinese Medicine consultations took place in 2009, of which acupuncture is a significant part of ref. 1. Often, related therapies, such as electro-acupuncture, auricular therapy, moxibustion, cupping, and transcutaneous electrical nerve stimulation (TENS) are also used in conjunction with acupuncture or on their own as a modality of treatment for various ailments and medical diseases. Traditional needle acupuncture has been gaining increasing popularity beyond China, as patients are becoming increasingly aware of and accepting of said acupuncture and its related modalities in the West. In the UK, approximately 4 million acupuncture sessions were offered to patients in 2009, with about two-third provided beyond the National Health Service^2^. In America, 3.1 million adults and 150,000 children used acupuncture in 2007^3^, which increased approximately by 1 million since 2002^4^. In 2003, 11% of Canadian adults sought help from complementary and alternative medicine (CAM), 2% of whom visited acupuncturists^5^.

In the UK, acupuncture and related therapies are most popular amongst patients with musculoskeletal pain and neurological conditions like headache and migraine^2^. Besides acupuncturists, it is also practiced by doctors, nurses, and physiotherapists with varying levels of training^2^. Although it is a common impression that acupuncture and related therapies are safe procedures, both minor and serious adverse events can occur during its use. Many publications have reported these adverse events but they are hard for clinicians to digest as they were written in inconsistent formats. The goal of this overview is to synthesize comprehensively existing systematic reviews on adverse events associated with acupuncture and related therapies, thus informing clinicians, acupuncturists, and patients alike on applying such treatments in a clinically mindful manner.

## Results

### Results on literature search and selection

**Figure 1:**
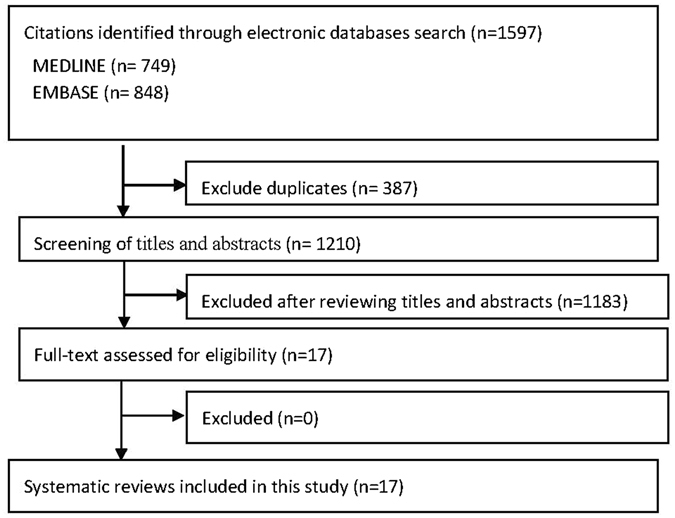
Flowchart of literature selection on systematic reviews of acupuncture related adverse events.

Electronic database search identified 1,597 citations, with 387 duplicates that were excluded before the screening process. Among the remaining 1,210 records, 1,183 were excluded based on title and abstract screening, leaving 17 publications for full text assessments. All of the 17 were found to be eligible and were included in the present overview. See Fig. 1 for the flow diagram of literature search and selection.

### Characteristics of included reviews

The 17 reviews were published between 1996 to 2015. The included reviews reported adverse events from a median of 43 primary studies (range, 9 to 167) and a median of 279 patients (range, 21 to 111,692). All 17 reviews were published in English. Seven (41.2%) reviews included only case reports, and the rest (10, 58.8%) included multiple study designs, including randomized or non-randomized clinical trials, cohort studies, case-control studies, cross-sectional studies, and case series. One review (5.9%) focused only on auricular acupuncture and related auricular therapies^6^, one (5.9%) focused only on electro-acupuncture^7^, six (35.3%) focused only on needle acupuncture^8–13^, three (17.6%) did not limit the type of acupuncture or related intervention that were included^14–16^, and the remaining six (35.3%) included two or more modalities, including needle acupuncture, electro-acupuncture, cupping, moxibustion, laser acupuncture, indwelling needles, dry needling, bee venom acupuncture, acupuncture point injection, and acupressure^17–22^. Four reviews (23.5%) included studies from China only^7, 10, 17, 19^, one (5.9%) included studies from the United States only^8^, one (5.9%) included studies from Norway only^13^, one (5.9%) included studies from Japan only^12^, and one (5.9%) included studies from the UK only^11^. The remaining reviews (9, 52.9%) included studies from two or more countries. See Table 1 for full details.

**Table 1:** Characteristics of included systematic reviews on acupuncture related adverse events.

First author and year of publication	Included study designs	Country of origin of included studies	Search period	Nature of acupuncture and related interventions	No. of studies (No. of patients) included
Norheim, 1996	Case reports	Norway	1981–1994	Needle acupuncture	78 (193)
Ernst, 2001	Prospective studies	5 countries in Europe, 4 countries in the Far East (Czechoslovakia, Sweden, Germany, Singapore, and Taiwan were reported)	Inception - 1999	Needle acupuncture, electro-acupuncture, cupping, moxibustion, laser acupuncture, or indwelling needles	9 (111, 692; including control group)
Yamashita, 2001	Case reports	Japan	1987–1999	Needle acupuncture	89 (124)
Ernst, 2003	Cohort, case-control, and cross sectional studies	United Kingdom	Inception – 2001	Needle acupuncture	15 (45922; including control group)
Lao, 2003	Case reports	United Kingdom, Germany, Belgium, United States, Israel, Ireland, New Zealand, Korea, Scotland, France, Norway, Japan, Taiwan, Australia, Canada, Brazil, Turkey, Italy, Spain, Nigeria, India, and China	1965–1999	Not stated	98 (202)
Bergqvist, 2008	Case reports	Not stated	Inception - NS	Needle acupuncture or electro-acupuncture	21 (21)
Zhang, 2010	Case reports and case series	China	1980–2009	Needle acupuncture	115 (479)
Adam, 2011	RCTs, cohort studies, case reports, and case series	Israel, China, France, Taiwan, Japan, United States, Canada, Hong Kong, Germany, United Kingdom	1950–2010	Needle acupuncture	37 (279)
Ernst, 2011a	Case reports and case series	Taiwan, Kapan, Hong Kong, China, Korea, Lima, United States, Canada, Malaysia, Australia, Croatia, Spain, France, Thailand, Ireland, United Kingdom, Spain, Holland, Singapore, Germany, and Switzerland	2000–2009	Needle acupuncture, dry needling, electro-acupuncture, bee venom acupuncture, laser acupuncture	94 (95)
Ernst, 2011b	Case reports and case series	China, Austria, Canada, France, India, Japan, Korea, Norway, and the United States	Inception - NS	Not stated	17 (26)
He, 2012	Case reports	China	1949–2010	Needle acupuncture, moxibustion, cupping, electro-acupuncture, laser acupuncture, auricular acupuncture, wrist and ankle acupuncture, acupuncture point injection, or acupressure	167 (1038)
Zheng, 2012	Case reports, case series, and surveys	China	1950–2010	Electro-acupuncture	15 (44)
Gnatta, 2013	No study design restrictions; all publications that reported infections caused by atypical mycobacteria after needle acupuncture were included	China, Spain, Portugal, South Korea, Canada, Venezuela, and Brazil	Inception - NS	Not stated	16 (406 including control group)
Xu, 2013	Case reports	Japan, Hong Kong, United Kingdom, United States, Ireland, Spain, Korea, France, Thailand, Australia, Canada, Malaysia, Taiwan, Croatia, Scotland, Venezuela, Brazil, Germany, Singapore, New Zealand, Iran, Austria, Greece, Italy, China, and Turkey	2000–2011	Needle acupuncture, moxibustion, or cupping	117 (308)
Tan, 2014	Case reports, case series, prospective and retrospective surveys, and all types of clinical trials	Australia, Austria, Canada, China, Germany, Hong Kong, Malaysia, Spain, Sweden, Taiwan, United Kingdom and United States	Inception - 2014	Auricular needle acupuncture, auricular electro-acupuncture, auricular acupressure, or auricular bloodletting therapy	43 (4203 including control group)
McCulloch, 2015	Case reports, case series, practice descriptions, and RCTs	United States	NS	Needle acupuncture	11 (>6000 including control group)
Wu, 2015	Case reports	China	1980–2013	Needle acupuncture or electro-acupuncture	133 (182)

### Methodological quality of included reviews

Fifteen (88.2%) reviews conducted a comprehensive literature search. No review provided an a priori design to their reviews via protocol publication. Thirteen (76.5%) review provided characteristics of their included studies. No review provided lists of both included and excluded studies. Eleven (64.7%) reviews reported the presence or absence of conflict of interests on the review itself. Only one (5.9%) review assessed the scientific quality of the included primary studies. The use of appropriate statistical methods and assessment on the likelihood of publication bias were not applicable as no meta-analysis was conducted amongst all included reviews. See Table 2 for full details.

**Table 2:** Methodological quality of included systematic reviews on acupuncture and its adverse events and/or complications.

First author and publication year	AMSTAR Item										
	1	2	3	4	5	6	7	8	9	10	11
Norheim, 1996	N	NR	N	NR	N	N	NR	N	NA	NA	N
Ernst, 2001	N	Y	Y	Y	N	Y	NR	Y	NA	NA	N
Yamashita, 2001	N	NR	N	N	N	N	NR	N	NA	NA	N
Ernst, 2003	N	Y	Y	Y	N	Y	NR	Y	NA	NA	N
Lao, 2003	N	NR	Y	N	N	N	NR	Y	NA	NA	Y
Bergqvist, 2008	N	NR	Y	Y	N	Y	NR	Y	NA	NA	N
Zhang, 2010	N	Y	Y	N	N	Y	NR	Y	NA	NA	Y
Adams, 2011	N	Y	Y	Y	N	Y	NR	Y	NA	NA	Y
Ernst, 2011a	N	NR	Y	Y	N	Y	NR	Y	NA	NA	Y
Ernst, 2011b	N	Y	Y	Y	N	Y	NR	N	NA	NA	Y
He, 2012	N	Y	Y	N	N	N	NR	N	NA	NA	Y
Zheng, 2012	N	Y	Y	NR	N	Y	NR	N	NA	NA	Y
Gnatta, 2013	N	NR	Y	Y	Y	Y	NR	Y	NA	NA	N
Xu, 2013	N	Y	Y	N	N	Y	NR	N	NA	NA	Y
Tan, 2014	Y	Y	Y	Y	N	Y	Y	Y	NA	NA	Y
McCulloch, 2015	N	Y	Y	NR	N	Y	NR	Y	NA	NA	Y
Wu, 2015	N	NR	Y	N	N	Y	NR	N	NA	NA	Y

Keys: Y, yes; N, no; NR, not reported; NA, not applicable. AMSTAR item: 1. Was an ‘a priori’ design provided? 2. Was there duplicate study selection and data extraction? 3. Was a comprehensive literature search performed? 4. Was the status of publication (i.e. grey literature) used as an inclusion criterion? 5. Was a list of studies (included and excluded) provided? 6. Were the characteristics of the included studies provided? 7. Was the scientific quality of the included studies assessed and documented? 8. Was the scientific quality of the included studies used appropriately in formulating conclusions? 9. Were the methods used to combine the findings of studies appropriate? 10. Was the likelihood of publication bias assessed? 11. Was the conflict of interest included?

### Adverse Events

Major results are narratively presented in this section and detailed information about the cases, including age and sex, reason for treatment, training background of practitioner, site of treatment, and follow-up time and outcome can be found in Table 3. A full list of results can be found in the Appendix 2.

**Table 3:** Most common adverse events and complications associated with acupuncture.

First author and publication year	Number of cases (age/sex)	Reason for acupuncture	Punctured Site	Practitioner	Follow-up Time and Outcome
**Organ or Tissue Injuries**					
***Pneumothorax***					
Norheim, 1996	23 (NS/NS)	NS	NS	Acupuncturists (at least half were conventional medical doctors)	NS
Yamashita, 2001	25 (NS/NS)	NS	NS	NS	NS
Lao, 2003	26 (NS/NS)	NS	Left nipple, above clavicle, back, intercostal space, parasternal, neck, paraspinal, chest below clavicle, supraclavicular, paravertebral, posterior aspect of shoulder, upper back, anterior chest, midthorax paraspine, chest, supraclavicular fossa, pericardial area	3 cases treated by non-medically trained acupuncturists, 1 chiropractor, 1 health clinic, 5 acupuncturists, 1 physician, 1 acupuncture office, 14 NS	23 recovered, 1 died, 2 NS
Zhang, 2010	201 (NS/NS)	NS	Shoulder, scapula, chest	NS	2–30 days, 197 recovered, 4 died
Ernst, 2011a	21 (25–72y/6M, 20F, 1 NS)	Pain (shoulder, neck, back), shoulder stiffness, algodystrophy, asthma, chronic cough, tension headache, 6 cases NS	NS	NS	17 recovered, 4 died
He, 2012	307 (NS/NS)	Periarthritis of the shoulders, cervical spondylosis, stiff neck, intercostal neuralgia, “others” (as reported by authors)	BL13, GB21, BL18, CV22, LI17, CV15, SI13, LI18, EX-B1, ST12, LU1, BL12, BL43, SP21, KI25	7 cases treated by acupuncturists, 23 private clinics, 4 barefoot doctors, 1 self, 272 NS	252 recovered, 6 died, 49 NS
Xu, 2013	13 (25–72y/3M, 10F)	Pain (back, shoulder, neck, musculoskeletal), asthma, stiff neck, breathing problem, chronic bronchitis, 1 case NS	Thoracic spine bilaterally, thoracic cavity, chest, upper back, back region, right scapular region, LU1, BL13, BL14, BL15, BL16	2 cases treated by acupuncturists, 1 acupuncture clinic, 1 medical acupuncturist, 1 registered TCM practitioner, 2 physiotherapists, 6 NS	2–12 days, 11 recovered, 1 died, 1 NS
Wu, 2015	30 (21–65y/13M, 17F)	Pain (shoulder, chest, back, cervical, neck, leg), gastroptosis, numbness of shoulders and chest, stiff neck, bronchitis, chronic bronchitis and emphysema, pulmonary heart disease, chronic asthmatic disease, hysteria, cough, spasmodic torticollis, scapulohumeral periarthritis, intercostal neuralgia, chronic hepatitis B, cervical spondylopathy	Shoulder, back, chest, supraclavicular fossa, scapular region, 6th intercostal space anterior axillar line, RN15, GB21, BL13, EX-B2, RN22, BL12, SI13, BL18, LI17, BL23, SI11, 2 NS	5 cases treated by acupuncturists, 3 factory doctors, 2 clinics, 1 hospital, 1 itinerant doctor, 3 individual clinics, 1 country doctor, 1 health center, 13 NS	3 days–1 month, 25 recovered, 2 died, 3 NS
***Central Nervous System Injury*** **/** ***Spinal Cord Injury***					
Norheim, 1996	13 (NS/NS)	NS	NS	NS	NS
Yamashita, 2001	25 (NS/NS)	NS	NS	NS	NS
Lao, 2003	13 (NS/NS)	Pain (lumbar, low back, neck, shoulder, cervical, posterior neck), neck stiffness, migraine, easily fatigued, bronchial asthma, stiff and painful shoulder, nervousness	NS	NS	8 recovered, 2 improved, 1 recovery of strength but not sensation, 2 sensory impairment remained
Zheng, 2012	2 (19–23y/2F)	Schizophrenia	GB20, GV15, GV14	NS	2 died
Xu, 2013	9 (29–74y/7M, 2F)	Pain (neck, lower back, quadi-paresis associated neck), stiff neck, 3 cases NS	Neck, lumbar region, upper back, posterior neck, GV16	3 cases treated by acupuncturists (1 unauthorized), 1 nonmedical practitioner, 1 oriental medicine clinic, 1 family physician, 3 NS	10 days–1 year, 8 recovered, 1 NS
Wu, 2015	37 (4.5–77y/21M, 16F)	Pain (neck, low back, lumbago, headache, migraine, toothache), impaired vision, schizophrenia, eyelid muscle twitch, bulbar palsy, headache, hysteria, chronic tracheitis, facial spasm, deaf-mute, psychosis, cerebral agenesis with apahasia, weakness of limbs, acid swells of the neck, aural vertigo, head stuffiness, facial paralysis, stiff neck, cold, neurosis, stroke, insomnia, cerebral hemorrhage, cervical spondylopathy, ankylosing spondylitis	Neck, posterior neck, back, waist, Ashi points near C3, T2-T3, GB20, DU16, DU14, DU15, EX-HN18, EX-HN21, LI11, LI4, GB30, ST36, GB39, GB14, ST2, BL37, KI01, RN16, RN10, RN9, RN4	NS	2–47 days, 23 recovered, 2 recovered after surgery, 12 died
***Subarachnoid*** **/** ***Intracranial Hemorrhage***					
Zhang, 2010	35 (NS/NS)	NS	GB20, GV15, GV16, GV14, BL10	NS	1–8 weeks, 32 recovered, 3 died
He, 2012	64 (NS/NS)	Headache, insomnia, neurasthenia, epilepsy, spasm of face	GB20, GV16, EX-HN14, HN13	1 case treated by acupuncturist, 63 NS	50 recovered, 12 NS, 2 died
McCulloch, 2015	4 (42–74y/NS)	NS	Scalp, GB20	NS	NS
***Cardiac Tamponade*** / ***Heart Injury***					
Bergqvist, 2008	7 (25–83y/2M, 5F)	NS	Breast, back, shoulder	NS	5 recovered, 2 congenital sternal foramen
Ernst, 2011b	26 (9–83y/6M, 8F, 12 NS)	Pain (chronic epigastric, back, multiple sites), fibromyalgia, intercostal neuralgia, chronic bronchitis, oesophagitis, dyspnea, diabetes, various, 7 cases NS	NS	NS	18 recovered, 6 died, 2 NS
Zheng, 2012	1 (30y/F)	Schizophrenia	CV15	NS	1 died
***Pseudoaneurysm***					
Bergqvist, 2008	7 (43–72y/5M, 2F)	Pain (abdominal and back), mass, pulsating mass, swelling, bleeding, fever	Shoulder, left knee, thigh, back, popliteal fossa, calf	NS	7 recovered
***Hematoma***					
Adams, 2011	44 (NS/NS)	Headache or chronic lower back pain or arthrosis	NS	44 cases treated by MDs trained in acupuncture	NS
***Broken needle*** **/** ***needle fragment***					
Ernst, 2011a	4 (29–70y/3M, 1F)	Dizziness, low back pain, sciatica, 1 case NS	NS	NS	4 recovered
***Soft-tissue injury***					
McCulloch, 2015	3 (60–82y/1M, 2F)	NS	NS	NS	NS
***Thumb deformity***					
Adams, 2011	12 (3–11y/4M, 8F)	NS	NS	NS	NS
**Infections**					
***Hepatitis***					
Norheim, 1996	100 (NS/NS)	NS	NS	NS	NS
Yamashita, 2001	11 (NS/NS)	NS	NS	NS	NS
Lao, 2003	94 (NS/NS)	NS	NS	36 cases treated by persons with no recognized medical qualification, 8 physicians practicing acupuncture, 6 chiropractic clinics, 35 licensed acupuncturists, 9 NS	42 recovered, 3 mild to severe liver failure, 3 chronic, 4 unknown, 1 died 41 NS
***Abscesses***					
Yamashita, 2001	6 (NS/NS)	NS	NS	NS	NS
Zhang, 2010	8 (19–52y/7M, 2F)	Tooth ache, gluteal numbness, insomnia, dizziness, headache, psoatic strain	Buccal, gluteal, cephalic, lower back	NS	8 recovered
Ernst, 2011a	8 (16–78y/5M, 3F)	No restriction of disease/symptoms (Pain (low back, hip, epigastric), weight loss, muscle strain, 1 case NS	NS	NS	8 recovered
Wu, 2015	4 (19–28y/2M, 2F)	Migraine, lumbar muscle strain	EX-HN5, GB14, DU20, Loin	1 case treated by health worker in the army, 1 NS	3–6 months, 4 recovered
***Tetanus***					
Zhang, 2010	2 (2F)	Pain (leg), headache	Cephalic, NS	NS	2 recovered
He, 2012	14 (NS/NS)	NS	NS	2 cases treated by barefoot doctors, 12 NS	5 recovered, 8 died, 1 NS
Wu, 2015	6 (2–62y/2M, 4F)	Pain (leg), malnutritional stagnation, headache, facioplegia, fever, rheumatoid arthritis	Knee, EX-UE19, EX-HN05, DU20, GB20, LI4, GB21, SJ5, 2 NS	2 cases treated by illegal treatment, 1 village acupuncturist, 1 private practitioner, 1 health-center, 1 NS	3 days–1 month, 3 recovered, 3 died
***Auricular Infections***					
Norheim, 1996	16 (NS/NS)	NS	NS	NS	NS
Lao, 2003	9 (NS/NS)	NS	NS	1 case treated by acupuncturist, 8 NS	4 residual deformities, 2 recovered, 3 NS
Adams, 2011	1 (16y/F)	Weight loss	NS	NS	NS
***Septic Arthritis***					
Adams, 2011	1 (13y/M)	Lumbar pain	NS	1 case treated by acupuncturist	NS
Ernst, 2011a	7 (13–78y/4F, 2M, 1 NS)	Pain (knee), rheumatoid arthritis, post-operative recovery, 1 case NS	NS	NS	7 recovered
***Local Infection***					
He, 2012	12 (NS/NS)	NS	NS	1 case treated by acupuncturist, 1 barefoot doctor, 10 NS	12 recovered
Tan, 2014	3 (NS/NS)	Smoking cessation, acute tonsillitis	Shenmen, lung, mouth, sympathetic, Ashi point	1 case treated by acupuncturist, 2 NS	NS
***Mycobacterial infection***					
Gnatta, 2013	295 (mean age 43–55y/162F, 33M, 100 NS)	NS	NS	NS	NS
Xu, 2013	193 (58–79y (4 participants, 189 NS)/3F, 1M, 189 NS)	Pain (ankle), knee osteoarthritis, obesity	GB38, abdomen, thigh, limb, 189 NS	NS	3 weeks to 4 months, 4 recovered, 189 NS
***Staphylococcal infection***					
Xu, 2013	29 (15–79 (11)/7M, 5F, 11 NS)	Pain (hip, low back, nuchal, knee and subscapular), postoperative recovery, shoulder stiffness, eczema	Low limb, around tibia, back, lower back, cervical paraspinal and medial scapular, shoulder and arm, hip, thigh, around the knee, bilateral paraspinal muscles	7 cases treated by acupuncturist, 1 practitioner, 1 TCM doctor, 20 NS	4 weeks–5 months, 12 recovered, 17 NS
**Local Adverse Events or Adverse Reactions**					
***Contact Dermatitis*** / ***Allergy***					
Norheim, 1996	3 (NS/NS)	NS	NS	NS	NS
Yamashita, 2001	2 (NS/NS)	NS	NS	NS	NS
Lao, 2003	7 (NS/NS)	NS	NS	NS	NS
Zhang, 2010	4 (11–52y/1M, 3F)	Cervical spondylosis, coxarthritis, abdominal pain	GB20, BL57, BL40, 1 NS	NS	4 recovered
Adams, 2011	11 (11–12y/1F, 10 NS)	Hip pain, emesis from general anaesthesia	NS	10 cases treated by anaesthesiologists trained by acupuncturist, 1 NS	NS
Ernst, 2011a	1 (65y/F)	Shoulder stiffness	NS	NS	NS
He, 2012	6 (NS/NS)	NS	NS	1 case treated by acupuncturist, 5 NS	6 recovered
Tan, 2014	~63 (NS/NS)	Post-operative pain, chronic low back pain, smoking cessation, insomnia, neurasthenia, obesity in female patients, vascular dementia, myopia, constipation, chemotherapy-induced nausea and vomiting, persistent allergic rhinitis, functional constipation	Shenmen, lung, mouth, brain, liver, kidney, heel, lesser occipital nerve, sympathetic, endocrine, heart, subcortex, stomach, spleen, pancreas, gallbladder, anterior ear lobe, San Jiao, great auricular nerve, hunger, colon, apex of ear, eye, large intestine, rectum, internal nose, wind stream, lumbar spine, cushion	2 cases treated by TCM practitioners, 2 acupuncturists, 1 therapist, 1 nurse, 57 NS	28 recovered, ~35NS
Wu, 2015	3 (54–72y/1M, 1F, 1 NS)	Scapulohumeral periarthritis, nerve root cervical spondylopathy, cervical type cervical spondylopathy	EX-UE01, GB21, LI11, SJ5, EX-B2	1 case treated by acupuncturist, 2 NS	10 minutes to 1 week, 3 recovered
***Argyria***					
Norheim, 1996	5 (NS/NS)	NS	NS	NS	NS
Yamashita, 2001	10 (NS/NS)	NS	NS	NS	NS
Ernst, 2011a	1 (66y/F)	Skin lesions	NS	NS	NS
Xu, 2013	1 (66y/F)	Arthralgia	Extremities	NS	NS
***Local Bleeding***					
Adams, 2011	~47 (10−<18y/1M, 1F, ~42NS)	Nocturnal enuresis, autism spectrum disorder, various, cerebral palsy, paralytic strabismus	NS	1 case treated by MD, ~40 acupuncturists, ~6 NS	NS
McCulloch, 2015	51 (NS/NS)	NS	NS	NS	NS
***Local Pain*** **/** ***Tenderness***					
Zhang, 2010	4 (NS/NS)	NS	SI3, LI11, hand	NS	4 recovered
Tan, 2014	~139 (NS/NS)	Drug dependence, chronic low back pain, obesity, pregnant women with low back pain and posterior pelvic pain, women with concurrent substance use problems and anxiety and depressive symptoms, substance abuse problem, smoking cessation, post-operative pain, alcohol withdrawal, drug use problem, psychological symptoms, physical discomfort in prison inmates	Shenmen, sympathetic, kidney, liver, lung, lumbar spine, cushion, stomach, hunger, endocrine, analgesia, mouth, knee joint, thalamus, hip joint, Ashi point, lumbosacral vertebrae, subcortex, unknown number of NS	8 cases treated by acupuncturists, 2 psychiatrists, 2 nurses, 1 physiotherapist, 121 NS	NS
***Local Burns***					
Lao, 2003	2 (NS/NS)	NS	NS	2 acupuncture clinics	NS
Zheng, 2012	1 (54y/M)	Leg pain	Right leg	NS	NS
He, 2012	1 (NS/NS)	NS	NS	NS	NS
Xu, 2013	2 (30, 32y/2M)	Pain (back)	Back	1 mother, 1 self	11 days, 2 recovered
***Local Bruising***					
McCulloch, 2015	1 (NS/NS)	NS	NS	NS	NS
**Other Complications**					
***Dizziness*** / ***Syncope***					
Norheim, 1996	2 (NS/NS)	NS	NS	NS	NS
Yamashita, 2001	1 (NS/NS)	NS	NS	NS	NS
Lao, 2003	2 (NS/NS)	NS	NS	1 acupuncturist	NS
Zhang, 2010	150 (30–57y/2M, 6F, 142NS)	Pain (low back, shoulder), stomach ache, cervical spondylosis, 146 cases NS	Shoulder, cervical, 148 NS	NS	150 recovered
He, 2012	468 (NS/NS)	NS	NS	194 cases treated by acupuncturists, 274 NS	394 recovered, 74 NS
Xu, 2013	3 (25–72y/2M, 1F)	Pain (arm, ankle), healthy volunteer for a clinical study	ST36, LI11, TB5, GB34, B40	NS	NS
Tan, 2014	~55 (48y, NS/1F, 51 NS)	Constipation, drug dependence, state anxiety before dental treatment, obesity, smoking cessation, post-operative pain, heroin addiction, cholecystolithiasis	Shenmen, kidney, liver, spleen, stomach, temple, subcortex, forehead, occiput, sympathetic, lung, relaxation, tranquilizer, master cerebral, hunger, endocrine, mouth, knee, joint, large intestine, rectum, San Jiao, extra, gallbladder, duodenum	1 case treated by physician, ~26 acupuncturists, 26 investigator with a diploma of acupuncture, unknown TCM practitioners 2 NS	2 withdrew from treatment, 3 recovered, ~50 NS
Wu, 2015	18 (24–78y/6M, 12F)	Pain (lumbago, shoulder, right thumb, stomache ache), facioplegia, myotenositis of long head of biceps brachii, scapulohumeral periarthritis, waist sprain, insomnia, prosopalgia, stroke, cervical spondylopathy, acute lumbar sprain, gouty arthritis, cervical spondylopathy radiculaire, facial paralysis	GB14, ST6, ST4, SJ17, LI20, LI14, LI15, LI11, Ashi, LI3, ST36, PC6, EX-UE01, GB21, LI14, LI11, SJ5, LI4, BL40, acupoint of bladder meridian, PC6, HT7, BL23, BL40, DU3, GB30, KI17, LI15, SJ5, SJ14, EX-HN5, SP6, EX-UE17, EX-LE11, BL10, GB20, SJ3, EX-B5, GB14, ST2, EX-HN16	18 cases treated by acupuncturists	10 minutes to 2 days, 18 recovered
***Nausea and vomiting***					
Lao, 2003	1 (NS/NS)	NS	NS	NS	1 recovered
Tan, 2014	~22 (NS/NS)	Drug dependence, smoking cessation, post-operative pain, heroin addiction, cholecystolithiasis	Shenmen, sympathetic, kidney, lung, liver, mouth, sympathetic, knee, joint, gallbladder, duodenum, stomach	~22 cases treated by acupuncturists, unknown number treated by TCM practitioners	1 recovered, ~21 NS
***Reduced bowel movements***					
Norheim, 1996	2 (NS/NS)	NS	NS	NS	NS
***Atrioventricular Block***					
Zheng, 2012	17 (NS/NS)	Psychosis	Between SJ17 and GB20	NS	17 recovered
***Epilepsy***					
Ernst, 2011a	2 (63–72y/1M, 1F)	Pain (forearm, ankle)	NS	NS	2 recovered
He, 2012	1 (NS/NS)	NS	NS	NS	NS
Wu, 2015	3 (35–53y/3M)	Soft tissue injury, epilepsy, cervical spondylosis	Ashi point, 2 NS	3 cases treated by acupuncturists	1 minute to 2 minutes, 3 recovered
***Factitial panniculitis***					
Ernst, 2011a	2 (22–24y/2F)	Weight loss	NS	NS	NS
***Aggravation of Bell’s Palsy***					
Zheng, 2012	13 (NS/NS)	Bell’s palsy	NS	NS	NS
***Galactorrhea***					
Xu, 2013	2 (32–41y/2F)	Pain (cancer, foot)	Upper back, foot	NS	NS
***Initial crying with fear and possible minor pain***					
Adams, 2011	~62 (NS/NS)	Cerebral palsy, autism spectrum disorder, persistent drooling	NS	~62 cases treated by acupuncturists	NS
***Vasovagal reaction***					
Adams, 2011	13 (NS/NS)	Headache, chronic lower back pain, arthrosis	NS	13 cases treated by MDs trained in acupuncture	NS
***Aphonia***					
Zhang, 2010	2 (36y, 46y/1M, 1F)	Hiccups	PC6	NS	2 recovered

Keys: NS, not stated; y, year; F, female; M, male; TCM, traditional Chinese medicine; MD, doctor of medicine; ~, approximately.

### Organ or tissue injuries (including complications from broken or remnant needles) associated with acupuncture

A median of 36 organ or tissue injuries were reported amongst 13 reviews^7–10, 12, 13, 15–20, 22^. A median of 4 deaths were reported across reviews, but four reviews did not report any data on outcomes^8, 9, 12, 13^. The most common organ or tissue injuries that occurred in patients included pneumothorax (8 reviews^10, 12, 13, 15, 17–19, 22^, median, 25.5, median number of deaths, 3), central nervous system or spinal cord injury (6 reviews^7, 12, 13, 16–18^, median, 13), subarachnoid or intracranial hemorrhage (3 reviews^8, 10, 19^, median, 35, median number of deaths, 2.5, with 1 review not providing outcome data^19^), and cardiac tamponade or heart injury (3 reviews^7, 15, 20^, median, 7, median number of deaths, 1). Respectively, one review reported each of the following as one of the most common organ or tissue injuries: pseudoaneurysm (7 cases)^20^, hematoma (44 cases)^9^, broken needle/needle fragment (4 cases)^22^, soft tissue injury (3 cases)^8^, and thumb deformity (12 cases)^9^. No deaths were reported for any of these complications. See Table 3 for full details.

### Infections associated with acupuncture

A median of 17 infections were reported amongst 11 reviews^6, 9, 10, 12–14, 16–19, 22^. A median of 0.5 deaths was reported, but 5 reviews did not report any data on outcomes^6, 9, 12–14^. The most common infections that occurred in patients included hepatitis (3 reviews^12, 13, 16^, median, 94, 1 death in total), abscesses (4 reviews^10, 12, 17, 22^, median, 7), tetanus (3 reviews^10, 17, 19^, median, 6, median number of deaths, 3), auricular infections (3 reviews^9, 13, 16^, median, 9), septic arthritis (2 reviews^9, 22^, median, 4), local infections (2 reviews^6, 19^, median, 7.5), Mycobacterial infection (2 reviews^9, 22^, median, 244), Staphylococcal infection (1 review^18^, 29 cases). See Table 3 for full details.

### Local adverse events or reactions associated with acupuncture

A median of 8.5 local adverse events or reactions were reported amongst 12 reviews^6–10, 12, 13, 16–19, 22^. No deaths were reported. The most common local adverse events or reactions that occurred in patients included contact dermatitis or local allergic reactions (9 reviews^6, 9, 10, 12, 13, 16, 17, 19, 22^, median, 4), argyria (4 reviews^12, 13, 18, 22^, median, 3), local bleeding (2 reviews^8, 9^, median, approximately 44), local pain or tenderness (2 reviews^6, 10^, median, approximately 71.5), local burns (4 reviews^7, 16, 18, 19^, median 1.5), ﻿and local bruising (1 review^8^, 1 case). See Table 3 for full details.

### Other complications associated with acupuncture

A median of 21 other complications were reported amongst 11 reviews^6, 7, 9, 10, 12, 13, 16–19, 22^. No deaths were reported. The most common other complications that occurred in patients included dizziness or syncope (8 reviews^6, 10, 12, 13, 16–19^, median, 10.5), nausea and vomiting (2 reviews^6, 16^, median, approximately 11.5), and epilepsy (3 reviews, median, 2). Respectively, one review reported each of the following as one of the most common other complications: reduced bowel movements (2 cases)^13^, atrioventricular block (17 cases)^7^, factitial panniculitis (2 cases)^22^, aggravation of Bell’s palsy (13 cases)^7^, galactorrhea (2 cases)^18^, initial crying with fear and possible minor pain (approximately 62 cases)^9^, vasovagal reaction (13 cases)^9^, and aphonia (2 cases)^10^. See Table 3 for full details.

## Discussion

This overview provided a comprehensive summary of all the adverse events and complications associated with acupuncture and related therapies that have been reported to date in published systematic reviews, with the majority coming from case reports, case series, and randomized controlled trials across 17 publications. The number of included primary studies in these publications ranged from 9 to 167. Amongst these primary studies, the number of included patients ranging from 21 to 111,692. In general, the results show that both minor and serious adverse events can occur from the use of acupuncture. Incidence rates, related confidence intervals (CIs), and p values could not be calculated because many adverse events came from case reports and many of the reviews did not include full details about the number of participants in their included studies. However, all the reviews have suggested that adverse events are rare and often minor.

Although serious complications were rare, they require significant attention as mortalities are associated with these adverse events. There was insufficient data to determine which body sites or whether patient predispositions were associated with these events, but it is clear that patients can be at great risk. Practitioners should pay ample attention to risk stratifying patients based on their medical history and other relevant characteristics. Other potential areas of improvement include enforcing stricter sterile needle practices, improving patient education about common and/or serious risks, and enhancing practitioner recognition of acute complications. Better communication should exist between physicians treating complications and the practitioner that administered the acupuncture, so that practitioners can become more cognizant of issues that can arise from their practice.

The methodological quality of the included reviews was mediocre. The majority of the included reviews conducted a comprehensive literature search. Most reviews also included the characteristics of their included studies and stated any conflicts of interest. However, no studies provided an a priori design, and only one study^6^ thoroughly assessed the scientific quality of included studies, which might be caused by lack of appropriate methodological quality assessment tools for case reports^23^. Regardless, the reviews provided a tantamount of information on the existence of adverse events in the literature across studies from around the world. A major limitation of the presented information was that no causality could be determined. In the reviews that commented on the scientific quality of included studies, concern was raised regarding the ability to ascertain that acupuncture resulted in the adverse outcome^8, 10, 11, 14, 22^. One review classified reported cases on a causality scale, and only a minority of adverse events were classified as certainly caused by acupuncture^19^. Future studies need to be more rigorous in their assessment of causality, and document their means of determining causality. Ideally, prospective cohort studies or randomized controlled trials should be reporting all the adverse events that occur during their investigations, as these provide the best evidence for causality. For rarer adverse events, case-control studies would be the ideal design. Another limitation is that a significant number of adverse events were not followed up. Although most complications were minor, practitioners and researchers should still follow up with the patient so that meaningful and definitive data can be derived. It is improper to assume that minor complications resolve spontaneously, or that major complications result in long-term implications. More attention needs to paid on the documentation and follow-up of all adverse events that occur during a study. Furthermore, a standardized template should be developed in the near future so that practitioners around the world can use it to track and report complications for research and clinical purposes.

Due to the diversity in study designs (e.g. case report, case series, case control studies, cohort studies, and clinical trials), populations, and data collection methods included in the identified systematic reviews, none of them conducted a meta-analysis to generate a pooled incidence rate with CI^24^. Although systematic reviews on adverse events are recommended to summarize evidence in a qualitative manner, a quantitative estimation of the upper limit of the 95% CI for the probability of the adverse events will help the clinicians to estimate what the worst-case scenario could be^24^. Future systematic reviews on this topic should use available statistical method^25^ to provide such a quantitative estimation. For primary studies, case control studies is the preferred method give the rare occurrence of adverse events.

Another concern raised by most reviews was the issue of underreporting^10, 11, 13, 15, 16, 19–21^. Often, only medically interesting findings are reported as many case reports are published by the physicians treating them^16^. Minor, less significant adverse events are often not published. Some reviews found that no adverse events were reported by acupuncturists^12^, raising the question of whether some practitioners are even aware of complications in their patients. Many journals restrict the type of publications that are accepted, and thus publication bias can also limit the number of adverse events reported^20^. This is a systemic issue, and regulatory bodies around the world need to create a convenient platform for which practitioners providing acupuncture and physicians treating complications can report known adverse events and the surrounding circumstances which can help with epidemiological and clinical research. In addition to providing a comprehensive summary, this overview serves as an important step towards furthering the knowledge, safety, and application of acupuncture. The overview may inform practitioners around the world about and modify the way they practice acupuncture, given that many acupuncturists may not be fully aware of the full breadth and depth of risk their treatments can pose. Usage of acupuncture is increasing worldwide, and more acupuncturists are being trained to match the demand. Thus, the need to pay careful attention towards the risks of acupuncture is becoming increasingly paramount as more patients become subject to it.

## Methods

### Inclusion criteria

Any systematic review (SR) that summarized adverse effects of acupuncture and related therapies, including electro-acupuncture, cupping, moxibustion, laser acupuncture, indwelling needles, dry needling, bee venom acupuncture, acupuncture point injection and acupressure, were considered eligible for this overview. To be included, the SR must have a primary objective of identifying adverse events instead of investigating its treatment efficacy or effectiveness. We had no restriction for the type of patients included, as long as they received acupuncture or related therapies for the management of any diseases or symptoms. We did not set any restriction on the control treatment as long as adverse effects of acupuncture were reported. However, SRs on adverse effects specifically caused by injected drugs through acupoint injections were excluded.

### Literature search

MEDLINE and EMBASE were searched from their inception to December 2015. Published search filters related to SR^26, 27^ and adverse effects^28^ were used during the literature search, in addition to search keywords for acupuncture and related therapies. Details on search strategies as well as the retrieved results from the electronic databases could be found in Appendix 1.

### Literature selection, data extraction and assessments of the methodological quality

Literature search was conducted by one researcher, and retrieved results were equally distributed to 4 pairs of trained research assistants. Each pair of assistants independently screened and evaluated the eligibility of citations that were assigned to them, and extracted data from the included reviews using a standardized, piloted template. The template was designed according to the requirement of the PRISMA harm checklist^29^. Disagreements were resolved via discussion and consensus within each pair. A senior researcher was consulted when disagreement was unresolvable.

Methodological quality of included SRs were evaluated with the validated Methodological Quality of Systematic Reviews (AMSTAR)^30^ instrument by two researchers independently. It includes 11 items, with each item being assessed as yes, no, cannot answer, or not applicable based on information provided by the SRs. Disagreements between assessors were discussed to reach consensus. A third reviewer was consulted if necessary.

### Data synthesis

Adverse effects of acupuncture and related therapies and their outcomes were narratively reported according to each adverse event. Protocol of this overview has been registered in PROSPERO (http://www.crd.york.ac.uk/PROSPERO/printPDF.php?RecordID=43943&UserID=6569).

## Electronic supplementary material

Supplementary Information
