# Supplementary material for: Safety of Acupuncture: Overview of Systematic Reviews
Source: Sci Rep. 2017 Jun 13;7:3369. doi: 10.1038/s41598-017-03272-0 (PMC5469776; doi:10.1038/s41598-017-03272-0)
Supplement: Supplementary file 1 — Supplementary Information [file 41598_2017_3272_MOESM1_ESM.pdf]

Safety of Acupuncture: Overview of Systematic Reviews

Malcolm WC Chan[1, 2 *], Xin Yin Wu[1, 3, 4 *], Justin CY Wu[1], Samuel YS Wong [3], Vincent CH Chung[1, 3, 4 #]

1. Hong Kong Institute of Integrative Medicine, The Chinese University of Hong Kong
2. Faculty of Medicine, University of Toronto
3. Jockey Club School of Public Health and Primary Care, The Chinese University of Hong Kong
4. Hong Kong Branch of the Chinese Cochrane Centre, The Chinese University of Hong Kong

* This two authors contributed equally to this work.

[#] Corresponding author: Dr Vincent CH Chung, 4/F, School of Public Health Building, Prince of Wales Hospital, Shatin, New Territories, Hong Kong; phone: (+852) 2252 8453; fax: (+852) 2145 7489; email: vchung@cuhk.edu.hk

Appendix 1. Search strategies and results

MEDLINE

1	MEDLINE.tw.	60960
2	systematic review.tw.	53458
3	meta analysis.pt.	62451
4	1 or 2 or 3	130578
5	exp acupuncture therapy/	18652
6	exp acupuncture, ear/	311
7	exp acupuncture points/	4621
8	exp acupuncture analgesia/	1105
9	exp acupuncture/	1371
10	acupunctur*.mp.	20070
11	exp electroacupuncture/	2876
12	electroacupunctur*.mp.	3528
13	acupoint*.mp.	2959
14	exp electric stimulation therapy/	63732
15	exp transcutaneous electric nerve stimulation/	6455
16	transcutaneous electric nerve stimulat*.mp.	3686
17	electric stimulation therap*.mp.	18033
18	percutaneous electrical nerve stimulat*.mp.	38
19	auriculoacupunctur*.mp.	9

20	TENS.mp.	7927
21	5 or 6 or 7 or 8 or 9 or 10 or 11 or 12 or 13 or 14 or 15 or 16 or 17 or 18 or 19 or 20	88205
22	Specific adverse effects*.mp.	148
23	(ae or co or de).fs.	5303611
24	(safe or safety or side effect* or undesirable effect* or treatment emergent or tolerability or toxicity or adrs or (adverse adj2 (effect or effects or reaction or reactions or event or events or outcome or outcomes))).ti,ab.	1018647
25	22 or 23 or 24	5818469
**26**	**4 and 21 and 25**	**749**

EMBASE

1	meta-analysis.tw.	92293
2	systematic review.tw.	81005
3	1 or 2	145922
4	exp acupuncture analgesia/	1419
5	exp acupuncture/	35834
6	exp acupuncture needle/	453
7	acupunctur*.mp.	35378
8	exp electroacupuncture/	4695
9	electroacupunctur*.mp.	5389
10	exp electrostimulation/	71299

11	exp electrostimulation therapy/	189714
12	electro-acupunctur*.mp.	933
13	acupoint*.mp.	4446
14	exp transcutaneous nerve stimulation/	6220
15	transcutaneous nerve stimulat*.mp.	6342
16	exp percutaneous electrical nerve stimulation/	6220
17	percutaneous electrical nerve stimulat*.mp.	68
18	TENS.mp.	11028
19	auriculoacupunctur*.mp.	13
20	4 or 5 or 6 or 7 or 8 or 9 or 10 or 11 or 12 or 13 or 14 or 15 or 16 or 17 or 18 or 19	290111
21	(safe or safety or side effect* or undesirable effect* or treatment emergent or tolerability or toxicity or adrs or (adverse adj2 (effect or effects or reaction or reactions or event or events or outcome or outcomes))).ti,ab.	1522396
22	Specific adverse effects*.mp.	211
23	21 or 22	1522396
**24**	**3 and 20 and 23**	**848**

Appendix 2. Other adverse events associated with acupuncture

Other adverse events associated with acupuncture

First author and publication year	Adverse event	Number of cases (age [y]/sex)	Reason for acupuncture	Punctured Site	Practitioner	Follow-up time
Norheim, 1996	Pseudoaneurysm	1 (NS/NS)	NS	NS	NS	NS
	Compartment Syndrome	1 (NS/NS)	NS	NS	NS	NS
	Deep venous thrombophlebitis	1 (NS/NS)	NS	NS	NS	NS
	Nerve injury	1 (NS/NS)	NS	NS	NS	NS
	Upper urinary tract injury	2 (NS/NS)	NS	NS	NS	NS
	Bacterial endocarditis	4 (NS/NS)	NS	NS	NS	NS
	Myelitis	3 (NS/NS)	NS	NS	NS	NS
	Staphylococcal sepsis	3 (NS/NS)	NS	NS	NS	NS
	HIV	1 (NS/NS)	NS	NS	NS	NS
	Psoas abscess	1 (NS/NS)	NS	NS	NS	NS
	Lymphocytoma cutis	1 (NS/NS)	NS	NS	NS	NS
	Asthmatic death	1 (NS/NS)	NS	NS	NS	NS
	Prurigo pigmentosa	1 (NS/NS)	NS	NS	NS	NS
	Reduced bowel	2 (NS/NS)	NS	NS	NS	NS

	movements					
	Hypotension/fainting	2 (NS/NS)	NS	NS	NS	NS
	Increased pain	1 (NS/NS)	NS	NS	NS	NS
	Behcets syndrome	1 (NS/NS)	NS	NS	NS	NS
	Eschars and scarring	1 (NS/NS)	NS	NS	NS	NS
	Problems from implanted needles	5 (NS/NS)	NS	NS	NS	NS
Ernst, 2001	Pre-collapse faintness	NS (NS/NS)	NS	NS	NS	NS
	Vasovagal collapse	NS (NS/NS)	NS	NS	NS	NS
	Needle fracture requiring surgical removal	NS (NS/NS)	NS	NS	NS	NS
	Aggravation of pain	NS (NS/NS)	NS	NS	NS	NS
	Abnormal tiredness	NS (NS/NS)	NS	NS	NS	NS
	Skin eruptions	NS (NS/NS)	NS	NS	NS	NS
	Edema	NS (NS/NS)	NS	NS	NS	NS
	Cyanosis with breathlessness	NS (NS/NS)	NS	NS	NS	NS
	Fatigue or exhaustion	NS (NS/NS)	NS	NS	NS	NS
	Aggravation of symptoms	NS (NS/NS)	NS	NS	NS	NS

	Failure to remove needle	NS (NS/NS)	NS	NS	NS	NS
	Ecchymosis or bleeding	NS (NS/NS)	NS	NS	NS	NS
	Burn injuries	NS (NS/NS)	NS	NS	NS	NS
	'Near syncope'	NS (NS/NS)	NS	NS	NS	NS
	Other vegetative symptoms (excluding dizziness, fainting)	NS (NS/NS)	NS	NS	NS	NS
Yamashita, 2001	Arterial injury	3 (NS/NS)	NS	NS	NS	NS
	Cardiac tamponade	2 (NS/NS)	NS	NS	NS	NS
	Renal injury	2 (NS/NS)	NS	NS	NS	NS
	Needle fragment(s) in the urinary tract	3 (NS/NS)	NS	NS	NS	NS
	Needle fragment(s) in the retroperitoneum	2 (NS/NS)	NS	NS	NS	NS
	Needle fragment(s) in the lung and the diaphragm	1 (NS/NS)	NS	NS	NS	NS
	Needle fragment(s) in the liver	1 (NS/NS)	NS	NS	NS	NS
	Needle fragment(s) in the maxilla	1 (NS/NS)	NS	NS	NS	NS
	Needle fragment(s) in the cervical interspinous	1 (NS/NS)	NS	NS	NS	NS

ligament					
Needle fragment(s) in the hip joint	1 (NS/NS)	NS	NS	NS	NS
Septicaemia	4 (NS/NS)	NS	NS	NS	NS
Spinal infection	4 (NS/NS)	NS	NS	NS	NS
Erysipelas	3 (NS/NS)	NS	NS	NS	NS
Streptococcal toxic shock-like syndrome	2 (NS/NS)	NS	NS	NS	NS, 1 death
Pyothorax or pyohemothorax	2 (NS/NS)	NS	NS	NS	NS, 1 death
Skull tuberculosis	1 (NS/NS)	NS	NS	NS	NS
Local redness	1 (NS/NS)	NS	NS	NS	NS
Subarachnoid hemorrhage	5 (NS/NS)	NS	NS	NS	NS
Medullary lesion	1 (NS/NS)	NS	NS	NS	NS
Epidural hematoma	1 (NS/NS)	NS	NS	NS	NS
Subdural hematoma	1 (NS/NS)	NS	NS	NS	NS
Peripheral nerve injury	3 (NS/NS)	NS	NS	NS	NS
Cutaneous chromatosis	1 (NS/NS)	NS	NS	NS	NS
Nodular lesion	2 (NS/NS)	NS	NS	NS	NS
Growth of tumour	1 (NS/NS)	NS	NS	NS	NS
Skin sarcoid	1 (NS/NS)	NS	NS	NS	NS
Subcutaneous bleeding	2 (NS/NS)	NS	NS	NS	NS

	Syncope	1 (NS/NS)	NS	NS	NS	NS
	Burn injury	1 (NS/NS)	NS	NS	NS	NS
Lao, 2003	Staphylococal septicemia	1 (NS/NS)	NS	NS	NS	NS
	Chronic osteomyelitis	1 (NS/NS)	NS	NS	NS	NS
	Spinal infection	1 (NS/NS)	NS	NS	1 osteopath	NS
	Staphylococcal septicemia	2 (NS/NS)	NS	NS	NS	NS, 2 deaths
	Bacterial endocarditis	3 (NS/NS)	NS	NS	1 NS, 1 nonmedically qualified acupuncturist, 1 natural healer	NS
	HIV infection	1 (NS/NS)	NS	NS	NS	NS
	Pseudoaneurysm	1 (NS/NS)	NS	NS	1 acupuncturist	NS
	Posterior tuberculous spondylitis	1 (NS/NS)	NS	NS	NS	NS
	Bacterial meningitis and epidural hematoma	1 (NS/NS)	NS	NS	1 in an unauthorized acupuncture clinic	NS
	Glenohumeral pyarthrosis	1 (NS/NS)	NS	NS	NS	NS
	Cervical spinal epidural abscess	1 (NS/NS)	NS	NS	NS	NS
	Peritemporomandibular abscess	1 (NS/NS)	NS	NS	NS	NS
	Cardiac tamponade	4 (NS/NS)	NS	Pericardial area,	1 self, 1	4 NS, 2 deaths

				sternum, level of fourth intercostal space (Ren 17), back of neck, shoulders, lower back, 1 NS	professional acupuncturist, 2 NS	
Renal lithiasis	1 (NS/NS)	NS		NS	NS	NS
Needle in left anterior chest well	1 (NS/NS)	NS		Lower abdominal wall	NS	NS
Needle in bladder	1 (NS/NS)	NS		Through urethra	1 self	NS
Foreign needle stone in ureter	1 (NS/NS)	NS		Left lumbar region	1 self	NS
Needles in soft tissue	1 (NS/NS)	NS		Between hip and T12	NS	NS
Multiple lymphocytoma cutis	1 (NS/NS)	NS		Auricular	NS	NS
Foreign body in kidney	1 (NS/NS)	NS		NS	NS	NS
Median nerve injury	1 (NS/NS)	NS		Dorsum of the wrist	NS	NS
Foreign body in ureter	1 (NS/NS)	NS		NS	NS	NS
Cardiac tamponade and hemothorax	1 (NS/NS)	NS		NS	1 self (professional acupuncturist)	NS
Factitial panniculitis	1 (NS/NS)	NS		Upper and lower extremities	NS	NS
Lipoatrophy	2 (NS/NS)	NS		Lateral aspect of upper arms	NS	NS
Lesion of the medulla oblongata	1 (NS/NS)	NS		NS	NS	NS
Drop foot	1 (NS/NS)	NS		Lower extremity	NS	NS

	Retroperitoneal hematoma	1 (NS/NS)	NS	Right loin region	1 physician	NS
	Post-traumatic sympathetic dystrophy	1 (NS/NS)	NS	NS	1 acupuncture clinic	NS
	Acute asthmatic attack	2 (NS/NS)	NS	NS	1 acupuncture clinic, 1 NS	2 NS, 1 death
	Bleeding	1 (NS/NS)	NS	NS	NS	NS
	Compartment syndrome	1 (NS/NS)	NS	NS	1 physician trained in acupuncture	NS
	Tingling in both hands and right arm	1 (NS/NS)	NS	NS	1 physician	NS
	Eschar and scarring	1 (NS/NS)	NS	NS	NS	NS
	Deep vein thrombophlebitis	1 (NS/NS)	NS	NS	1 acupuncturist	NS
	False aneurysm of the popliteal artery	1 (NS/NS)	NS	NS	NS	NS
	Petechiae	1 (NS/NS)	NS	NS	1 acupuncturist	NS
	Cutaneous herpes	1 (NS/NS)	NS	NS	NS	NS
	Electromagnetic interference with pacemaker	1 (NS/NS)	NS	NS	NS	NS
	Hypotension	1 (NS/NS)	NS	NS	1 physician	NS
Bergqvist, 2008	Popliteal artery occlusion	1 (44/F)	NS	NS	NS	NS
	Deep vein thrombosis	1 (64/F)	NS	NS	NS	NS
	Aortoduodenal	1 (68/M)	NS	NS	NS	NS

	fistula					
	Thrombophlebitis of the left crural vein	1 (NS/NS)	NS	NS	NS	NS
	Compartment syndrome	1 (NS/NS)	NS	NS	NS	NS
Zhang, 2010	Spinal epidural haematoma	9 (NS/NS)	NS	GB20, GV15, GV16, GV14, BL10	NS	NS
	Chylothorax	1 (NS/NS)	NS	BL13	NS	NS
	Right ventricular injury	4 (NS/NS)	NS	NS	NS	NS, 2 deaths
	Aortic artery rupture	1 (NS/NS)	NS	LR14	NS	NS, 1 death
	Coronary artery injury with cardiac tamponade	1 (NS/NS)	NS	LU1	1 self	NS
	Injuries of abdominal organs and tissues (including but not exclusive to gallbladder perforation, bowel perforation, stomach perforation, peritonitis	16 (NS/NS)	NS	ST25, CV12, LR14	NS	NS
	Intestinal wall haematoma	1 (2/M)	NS	ST25, CV12, LR14	NS	NS
	Neural injuries	4 (NS/NS)	NS	NS	NS	NS
	A false aneurysm of the carotid artery	1 (NS/NS)	NS	NS	NS	NS
	Thyroid haemorrhage	1 (NS/NS)	NS	NS	NS	NS

Death from neck acupuncture	1 (NS/NS)	NS	CV22	NS	NS, 1 death
Orbital haemorrhage	3 (NS/NS)	NS	SL1, EX-HN7, ST1	NS	NS
Traumatic cataract	1 (NS/NS)	NS	SL1, EX-HN7, ST1	NS	NS
Oculomotor nerve	1 (NS/NS)	NS	SL1, EX-HN7, ST1	NS	NS
Retinal puncture	1 (NS/NS)	NS	SL1, EX-HN7, ST1	NS	NS
Optic atrophy with haemorrhage and traumatic cataract	1 (NS/NS)	NS	SL1, EX-HN7, ST1	NS	NS
Haemorrhage	3 (NS/NS)	NS	Cheek, hypoglottis	NS	NS
Calf haematoma complicated by diabetic foot	1 (NS/NS)	NS	ST38, BL57	NS	NS
Peripheral motor nerve injury and subsequent motor dysfunction	4 (NS/NS)	NS	EX-HN5, PC6, lI4	NS	NS
Adductor muscle fibrosis and adduction deformity of the thumb due to local vascular and muscular injuries	3 (NS/NS)	NS	L14, EX-HN5, PC6, lI4	NS	NS
Suppurative arthritis	1 (38/M)	Arthritis	EX-LE5	NS	NS
Stroke / cerebral haemorrhage	5 (58-73/4M, 1F)	Arm pain, rheumatoid arthritis, stroke-related hemiplegia, sequela of cerebral haemorrhage, sequela of stroke	LI4, LI10, LI11, SJ3, GB20, CV23, LI4, LI11, ST36, KI1, EX-HN5, GV20	NS	NS, 3 deaths
Cardiac arrest	1 (28/M)	Tic douloureu	EX-HN5, LI4, SJ5	NS	NS
Shock	1 (39/M)	Asthma	BL13	NS	NS

	Pyknolepsy	1 (35/M)	Cheiralgia	Painful spot	NS	NS
	Orbital haemorrhage	1 (46/F)	Cold, headache	EX-HN5	NS	NS
	Fever	1 (52/M)	Sequela of brain concussion	GV20, GB20, LI11, LI4	NS	NS
	Cough	1 (65/F)	Facioplegia	GB14, LI4	NS	NS
	Thirst	1 (46/F)	Obesity	ST25, SP15	NS	NS
	Leg numbness	1 (47/F)	Sciatica	SI3	NS	NS
	Sexual dysfunction	2 (52, 41/1M, 1F)	Simple obesity	CV4	NS	NS
	Spermatorrhea	1 (20/M)	Headache	KI1	NS	NS
Adams, 2011	Hospitalization for emesis	1 (NS/NS)	Emesis related to dental anaesthesia	NS	1 MD with TCM license	NS
	HIV infection	1 (17/M)	Tendonitis	NS	NS	NS
	Septic sarcoiliitis	1 (14/F)	Mild pain in right buttock	NS	NS	NS
	Pyogenic spondylitis	1 (15/M)	Thoracic spinal pain	NS	NS	NS
	Pott’s puffy tumor	1 (12/F)	Neurologic condition	NS	1 acupuncturist	NS
	Cardiac rupture	1 (9/M)	Tuberculosis, heart disease	NS	1 acupuncturist	NS
	Nerve impairment	1 (16/M)	Tiredness, rapid heartbeat, constipation	NS	NS	NS
	Subarachnoid hemorrhage	1 (11/F)	Limited speech and hearing abilities	NS	1 acupuncturist	NS
	Intestinal obstruction	1 (2/M)	Diarrhea	NS	NS	NS
	Hemoptysis from aspirated needle	1 (15/M)	Encephalopathy	NS	NS	NS
	Reversible coma	1 (15/M)	Musculoskeletal pain	NS	1 acupuncturist	NS

	Numbness	3 (NS/NS)	Persistent allergic rhinitis	NS	1 acupuncturist	NS
	Headache	1 (NS/NS)	Persistent allergic rhinitis	NS	1 acupuncturist	NS
	Light-headedness	1 (NS/NS)	Persistent allergic rhinitis	NS	1 acupuncturist	NS
	Needle Pain	5 (NS/NS)	Emesis from chemotherapy	NS	5 acupuncturists	NS
	Fainting	2 (NS/NS)	NS	NS	NS	NS
	Pain on insertion	~6 (NS/NS)	NS	NS	~6 acupuncturists	NS
	Petechiae or ecchymosis	~6 (NS/NS)	NS	NS	~6 acupuncturists	NS
	Pneumothorax	1 (15/F)	Asthma	NS	1 acupuncturist	NS
	Vasovagal reaction	13 (NS/NS)	Headache or chronic lower back pain or arthrosis	NS	13 MDs trained in acupuncture	NS
	Drowsiness/sleep disturbance	1 (NS/NS)	Headache or chronic lower back pain or arthrosis	NS	1 MD trained in acupuncture	NS
	Aggravation of condition	5 (NS/NS)	Headache or chronic lower back pain or arthrosis	NS	5 MDs trained in acupuncture	NS
	Sensation during treatment	1 (NS/NS)	Headache or chronic lower back pain or arthrosis	NS	1 MD trained in acupuncture	NS
	Hemorrhage	4 (NS/NS)	Autism spectrum disorder	NS	1 acupuncturist	NS
	Petechiae, multiple	1 (2/F)	Pneumonia	NS	1 acupuncturist	NS
	Prolonged migraine	1 (16/M)	Migraine	NS	1 MD trained in acupuncture	NS
	Worsened symptoms	1 (15/F)	Pain related to spina bifida	NS	NS	NS
Ernst, 2011a	Pott puffy tumour with pseudomonas infection	1 (12/F)	Neurologic sequelae of encephalitis	NS	NS	NS
	Pneumoretroperiton	1 (25/F)	Low back pain	NS	NS	NS

eum					
Necrotizing aortitis	1 (79/M)	Low back pain	NS	NS	NS
E. coli sepsis	1 (64/M)	Low back pain	NS	NS	NS
Diabetic ketoacidosis	1 (31/M)	Muscle strain, hip pain	NS	NS	NS
Cervical subdural haematoma	1 (44/M)	Neck pain	NS	NS	NS
Spondylodiscitis caused by staphylococcus	1 (50/M)	Low back pain	NS	NS	NS
Necrotizing fasciitis	1 (55/F)	Osteoarthritis of the knee	NS	NS	NS
Staphylococcus aureus sepsis, pleural empyema, polyarthritis, left hand phlegmon	1 (53/F)	Shoulder stiffness	NS	NS	NS
Mycobacterium chelonae infection	1 (58/F)	Weight loss	NS	NS	NS
Facial erysipelas	1 (70/F)	NS	NS	NS	NS
Syphilis infection	1 (62/F)	Arthritis	NS	NS	NS
Infection with HIV-virus	1 (60/F)	Muscle pain	NS	NS	NS
S. aureus infection	1 (73/M)	Low back pain	NS	NS	NS
S. aureus septicaemia	1 (33/M)	Hip pain	NS	NS	NS
Infected compartment syndrome	1 (37/M)	Tendonitis	NS	NS	NS
Mycobacterial	1 (79/F)	Chronic pain	NS	NS	NS

infection (right leg)					
Mycobacterial infection (left ankle)	1 (21/M)	Lupus erythematosus	NS	NS	NS
Mycobacterial infection (right wrist)	1 (62/F)	Dizziness	NS	NS	NS
Mycobacterial infection (left wrist)	1 (72/M)	Chronic pain	NS	NS	NS
Prosthetic valve endocarditis	1 (42/F)	Low back pain	NS	NS	NS
Soft tissue infection with mycobacterium chelonae	1 (79/F)	Knee osteoarthritis	NS	NS	NS
Pyoderma gangrenosum	1 (48/F)	Arthritis	NS	NS	NS
Bilateral hand oedema	1 (52/M)	Low back pain	NS	NS	NS
Infectious aortic aneurysm	1 (67/M)	NS	NS	NS	NS
Pneumomediastinum	1 (57/F)	Tension headache	NS	NS	NS
Median nerve neuropathy	1 (47/M)	Abdominal pain	NS	NS	NS
Haemorrhage in carpal tunnel	1 (81/F)	Stiffness of finger	NS	NS	NS
Injury of cervical spinal cord	1 (31/F)	Neck pain	NS	NS	NS
Pleural empyema	1 (35/F)	Low back pain	NS	NS	NS
NS	2 (32, 41/2 F)	Sudden bilateral headache, vomiting, cancer pain	NS	NS	NS

Popliteal artery pseudoaneurysm	1 (71/M)	Intermittent claudication	NS	NS	NS
Temporomandibular joint pain	1 (47/M)	Intermittent claudication	NS	NS	NS
Epidural haematoma	1 (30/M)	Back pain	NS	NS	NS
Injury of arteria brachialis, ischemia of hand	1 (47/F)	Colitis ulcerosa	NS	NS	NS
Aorta-duodenal fistula, shock	1 (68/M)	Low back pain	NS	NS	NS
Rectus sheath haematoma	1 (37/F)	Weight loss	NS	NS	NS
Cardiac tamponade	3 (25, 49, NS/2 F, 1 NS)	Shoulder pain, abdominal pain, 1 NS	NS	NS	NS
Acute traumatic pancreatitis (due to puncture of pancreas by long acupuncture needle)	1 (42/F)	Dyspepsia	NS	NS	NS
Pseudoaneurysm of abdominal aorta	1 (54/M)	Abdominal pain	NS	NS	NS
Intracranial haemorrhage	1 (44/M)	Neck pain	NS	NS	NS
Cerebrospinal fluid fistula	1 (52/M)	Low back pain	NS	NS	NS
Nystagmus, vertigo, hypotension	1 (50/F)	Shoulder pain	NS	NS	NS
Galactorrhoae of left breast	1 (NS/F)	Chronic pain in left hallux	NS	NS	NS

	Granuloma compressing lumbar 4th nerve	1 (68/F)	Low back pain	NS	NS	NS
	Basal cell carcinoma of earlobe	1 (65/F)	NS	NS	NS	NS
	Pseudolymphoma	1 (37/F)	Abdominal pain	NS	NS	NS
	Behcet disease	1 (35/M)	Tennis elbow	NS	NS	NS
	Trigeminal neuralgia	1 (65/F)	Shoulder stiffness	NS	NS	NS
	Silicone granuloma, suspected sarcoidosis	1 (55/F)	Musculoskeletal pain	NS	NS	NS
He, 2012	Hemorrhage and hematoma	22 (NS/NS)	NS	NS	3 acupuncturists, 19 NS	NS
	Nerve injury	10 (NS/NS)	NS	NS	NS	NS
	Death	8 (NS/NS)	NS	NS	1 acupuncturist, 2 barefoot doctors, 5 NS	NS
	Epidural hematoma	7 (NS/NS)	NS	NS	NS	NS
	Heart rupture and death	3 (NS/NS)	NS	NS	2 private clinics, 1 barefoot doctor	NS, 3 deaths
	Intestinal tube injury	2 (NS/NS)	NS	NS	NS	NS
	Other organ or tissue injury	28 (NS/NS)	NS	NS	2 acupuncturists, 1 private clinic, 1 barefoot doctor, 20 NS	NS, 7 death
	Peritonitis	7 (NS/NS)	NS	NS	3 self, 4 NS	NS
	Sepsis	1 (NS/NS)	NS	NS	NS	NS

Intracranial infection	1 (NS/NS)	NS	NS	NS	NS
Deep vein inflammation	1 (NS/NS)	NS	NS	NS	NS
Osteomyelitis	1 (NS/NS)	NS	NS	NS	NS
Facial neuritis	1 (NS/NS)	NS	NS	NS	NS
Forgotten the needle	19 (NS/NS)	NS	NS	NS	NS
Breaking of the needle	15 (NS/NS)	NS	NS	1 private clinic, 1 self, 13 NS	NS
Bending of the needle	13 (NS/NS)	NS	NS	NS	NS
Sticking of the needle	7 (NS/NS)	NS	NS	NS	NS
Fever	1 (NS/NS)	NS	NS	NS	NS
Lame foot	1 (NS/NS)	NS	NS	NS	NS
Stomache ache	1 (NS/NS)	NS	NS	NS	NS
Hyperventilation syndrome	1 (NS/NS)	NS	NS	NS	NS
Allergic shock	1 (NS/NS)	NS	NS	NS	NS
Cough	1 (NS/NS)	NS	NS	NS	NS
Thirst	1 (NS/NS)	NS	NS	NS	NS
Likeness of infusion reaction	1 (NS/NS)	NS	NS	NS	NS
Abortion	1 (NS/NS)	NS	NS	NS	NS
Scald of skin	1 (NS/NS)	NS	NS	NS	NS
Hoarseness	1 (NS/NS)	NS	NS	NS	NS

	Blindness	1 (NS/NS)	NS	NS	NS	NS
	Retinal detachment	1 (NS/NS)	NS	NS	NS	NS
	Shock	1 (NS/NS)	NS	NS	NS	NS
	Subcutaneous hemorrhage	1 (NS/NS)	NS	NS	NS	NS
	Subcutaneous emphysema	1 (NS/NS)	NS	NS	NS	NS
	Subcutaneous scleroma	1 (NS/NS)	NS	NS	NS	NS
	Nettlerash	1 (NS/NS)	NS	NS	NS	NS
	Discomfort	1 (NS/NS)	NS	NS	NS	NS
	Fat liquefaction	1 (NS/NS)	NS	NS	NS	NS
Zheng, 2012	Hydropneumothorax	1 (35/F)	Hysteria	Supraclavicular fossa	NS	NS
	Tardive fainting	1 (42/M)	Lumbar strain	EX-B2, BL40	NS	NS
	Shock	1 (42/F)	Carcinoma of thyroid	SJ17	NS	NS
	Hyperventilation syndrome	1 (35/F)	Nape pain	GB20, EX-B2	NS	NS
	Nerve injury	1 (53/F)	Trigeminal neuralgia	Trigeminal nerve ramus of mandible	NS	NS
	Femoral-neck fracture	1 (40/M)	Schizophrenia	EX-HN8	NS	NS
	Subluxation of wrist joint	1 (65/F)	Hemiplegia	LI15, SJ5, LI4	NS	NS
	Nape muscle spasm	1 (17/F)	Myopia	GB20, EX-HN14	NS	NS
	Hemorrhagic shock	1 (61/M)	Rheumatic arthritis	ST34, SP10, GB33, GB34, ST40, SP6	NS	NS
	Gastric ulcer complicated by	1 (54/M)	Gastric ulcer	ST36	NS	NS

	perforation					
	Oculomotor paresis	1 (NS/NS)	NS	NS	NS	NS
Xu, 2013	Infectious aortic aneurysm	1 (67/M)	NS	NS	NS	>- 8 days
	Septic arthritis	1 (13/M)	Low back pain	NS	1 acupuncturist	1 weeks
	Endocarditis	1 (42/F)	Low back pain	NS	NS	3 weeks
	Streptococcus	1 (37/M)	Tendonitis	BL57	NS	NS
	Infected left atrial myxoma (Gram-positive)	1 (47/F)	Weight loss	Earlobes	NS	After surgery
	Klebsiella pneumoniae	1 (56/M)	Right flank discomfort	NS	NS	NS
	Facial erysipelas	1 (70/F)	NS	Face	1 physiotherapist	4 weeks
	HIV	1 (60/F)	Muscle pain	NS	1 non-MD	NS
	Tissue abscess and osteomyelitis	1 (44/F)	NS	Thigh	NS	NS
	Septic arthritis	1 (48/F)	Low back pain	Low back	NS	3 weeks
	Intra-abdominal abscess	1 (64/F)	Epigastric pain	Abdomen (embedded needles)	NS	3 weeks
	Necrotizing fasciitis	3 (44-84/1M, 2F)	Knee osteoarthritis, osteoarthritis, calf pain	Knee, left groin, calf	1 TCM doctor, 2 NS	1 NS, 1 2 months
	Escherichia Coli	1 (64/M)	Low back pain	Lumbar paraspinal muscles	NS	NS
	Cellulitis, septicemia, and pneumonia	1 (69/M)	Knee pain	SP10	NS	NS
	Listeria monocytogenes	1 (78/M)	Knee rheumatic arthritis	Knee	1 acupuncturist	3 weeks

septic arthritis					
Pseudomonas aeruginosa	1 (16/F)	Weight loss	Auricular	1 acupuncture parlor	21 days
Escherichia coli	1 (79/M)	Low back pain	Back	NS	76 days
Pneumoretroperiton eum	2 (25/F)	Low back pain	Back, 1 NS	2 OMD	1 week
Factitial panniculitis	3 (22-56/, 3 F)	Weight loss, 1 NS	Both arms, abdomen, neck, and shoulder	1 self, 2NS	3 months (1), 2 NS
Pott’s puffy tumor from pseudomonas	1 (12/M)	Neurologic sequelae of encephalitis	Head	NS	8 weeks
NTM	1 (23/F)	NS	NS	NS	6 months
Enterococcus faecalis knee infection	1 (60/F)	Knee pain	Needles embedded at knee	NS	1 year
Auricular cellulitis	2 (21, 30/1M, 1F)	Obesity	Auricular	2 acupuncturists	2 days – 1 week
Psoas abscess	1 (53/F)	Low back pain	Lower back	NS	2 weeks
Cutaneous tuberculosis infection	3 (72-77/1M, 2F)	NS	Abdomen, thigh, back, shoulder, and right thigh	1 illegal treatment	9 months – 1 year
Peroneal nerve palsy	1 (62/F)	Sciatica	Anterior of the leg	NS	4 months
Injury of the L5 nerve root	1 (63/F)	Low back pain	Low back	NS	NS
Bell’s Palsy	1 (47/M)	TMD	ST6, ST7	NS	2 weeks
Median nerve neuropathy	1 (47/M)	Abdominal discomfort	PC5, PC6	1 oriental medicine practitioner	1 year
Cardiac tamponade	2 (49, 83/2F)	NS	Sternum	1 experienced acupuncturist, 1	2 weeks (1), 1 NS

				NS	
Right ventricular embolism	1 (70/M)	Chronic lung disease	Neck, chest, and abdomen	NS	NS
Myocardium injury	1 (69/F)	Pain	Shoulders and neck (implanted needles)	1 traditional medicine practitioner	NS
Hemopericardium	1 (54/F)	Myalgia and dyspepsia	Chest, abdomen	1 ynauthorized acupuncturist	6 days
Pseudoaneurysm of abdominal aorta	1 (54/M)	Abdominal pain	Back	1 OMD	8 days
Pseudoaneurysm of the popliteal artery	1 (61/F)	Osteoarthritis	Knee	NS	1 year
Acute traumatic pancreatitis	1 (42/F)	Dyspepsia	Abdomen	1 acupuncture clinic	4 days
Aortoduodenal fistula	1 (68/F)	Low back pain	Abdomen	NS	NS, 1 death
Rectus sheath hematoma	1 (37/F)	Weight loss	Abdomen	NS	1 month
Ear hematomas	1 (78/M)	Postoperative pain	Ear lobe	NS	NS
Popliteal arteriovenous fistula	1 (39/F)	Knee soreness	Popliteal fossa	NS	NS
Bilateral hand edema	1 (52/M)	Low back pain	LI4	NS	8 weeks
Epithelioid granuloma at needling sites	1 (55/F)	Shoulder pain and lumbago	Back, hip, neck, legs and arms	NS	NS
Pseudolymphoma	1 (37/F)	Abdominal discomfort	NS	NS	NS
Pustules	1 (35/M)	Tennis elbow	Arm	NS	NS
Pancytopenia	1 (67/F)	Low back pain	Chest and abdomen	NS	NS

Scars at needling site	1 (36/F)	Hyperthyroidism	ST10	NS	NS
Pyoderma gangrenosum	1 (48/F)	Arthralgia	NS	NS	NS
Nystagmus	1 (50/F)	Shoulder pain	Points around shoulder	NS	NS
Spontaneous needle movement	1 (55/M)	Back pain	Back	NS	NS
Hepatotoxicity	1 (52/F)	Leg weakness	ST36	NS	NS
Eruptive lichen planus	1 (41/F)	Back pain	Lower back	NS	NS
Ecchymoses	1 (38/M)	NS	Abdomen	NS	NS
Cellulitis	1 (53/F)	Headache	Leg and feet	1 untrained individual	NS
Infection caused by spinal epidural abscess	1 (78/F)	Pain	Fingers	1 self	NS
Basal cell carcinoma	1 (58/M)	Abdominal pain	Abdomen	1 self	NS
Keloid scar	1 (36/F)	Cough	Back	NS	Several days
Suction bullae	1 (57/F)	Low back pain	Low back	NS	NS
Acquired hemophilia A	1 (58/F)	NS	Thigh	NS	1 week
Reversible cardiac hypertrophy	1 (66/F)	Pain	NS	1 self	3 months
Iron deficiency anemia	1 (39/M)	Musculoskeletal pain	Back	NS	NS
Bullae	1 (55/M)	NS	Back	NS	Several weeks
Hemorrhagic stroke	1 (55/M)	NS	Back and neck	NS	NS

	Burns on back and shoulder	1 (32/M)	Back pain	Back	1 mother	NS
Tan, 2014	Somnolence	2 (41, 43/2M)	Lumbar muscle strain, dilated cardiomyopathy	Heart, liver, spleen, occiput, shenmen, sympathetic, lung	NS	NS
	Abdominal pain	1 (58/F)	Chronic diarrhea	Large intestine, small intestine, shenmen, liver, sympathetic, spleen	NS	NS
	Uncomfortable at needling site	1 (NS/NS)	PTSD-related insomnia	Shenmen, liver, kidney, sympathetic, hippocampus	1 physician	NS
	Light headache	17 (NS/NS)	Drug dependence	Shenmen, sympathetic, kidney, liver, lung	17 acupuncturists	NS
	Slight bleeding	14 (NS/NS)	Drug dependence	Shenmen, sympathetic, kidney, liver, lung	14 acupuncturists	NS
	Tingling sensations	19 (NS/NS)	Drug dependence	Shenmen, sympathetic, kidney, liver, lung	19 acupuncturists	NS
	Drowsiness	11 (NS/NS)	Drug dependence	Shenmen, sympathetic, kidney, liver, lung	11 acupuncturists	NS
	Dry mouth	15 (NS/NS)	Drug dependence	Shenmen, sympathetic, kidney, liver, lung	15 acupuncturists	NS
	Slight fever	19 (NS/NS)	Drug dependence	Shenmen, sympathetic, kidney, liver, lung	19 acupuncturists	NS
	Swelling around needling site	1 (NS/NS)	Chronic low back pain	Shenmen, lumbar spine, cushion	1 physiotherapist	NS

Warmth or a strange feeling at the treated ear	26 (NS/NS)	State anxiety before dental treatment	Relaxation, tranquilizer, master cerebral	26 investigators with a diploma of acupuncture	NS
Transient exacerbation of vasomotor symptoms	2 (NS/NS)	Hot flushes in prostate caner patients with LHRH agonist treatment	Shenmen, liver, lung, autonomic, kidney	NS	NS
Hematoma	1 (NS/NS)	Smoking cessation	Shenmen, lung, mouth, sympathetic	1 acupuncturist	NS
Feeling of residual needling	24 (NS/NS)	Smoking cessation	Shenmen, lung, mouth, sympathetic	24 acupuncturists	NS
Minor bleeding	3 (NS/NS)	Smoking cessation	Shenmen, lung, mouth, sympathetic	3 acupuncturists	NS
Pain at insertion and sleep disturbance	1 (NS/NS)	Postoperative pain	Shenmen, lung, knee joint	1 acupuncturist	NS
Mild bleeding	6 (NS/NS)	Alcohol withdrawal	Shenmen, sympathetic, kidney, liver, lung	6 psychiatrists or mental-health nurses	NS
Minor bleeding at needling site	2 (NS/NS)	Postoperative pain	Shenmen, lung, thalamus, hip joint	2 acupuncturists	NS
Headache	1 (NS/NS)	Postoperative pain	Shenmen, lung, thalamus, hip joint	1 acupuncturist	NS
Hip pain after needle withdrawal	2 (NS/NS)	Postoperative pain	Shenmen, lung, thalamus, hip joint	2 acupuncturists	NS
Infrequent minor bleeding upon needle removal	NS (NS/NS)	Smoking cessation and cigarette consumption	Shenmen, lung, liver, kidney, sympathetic	1 acupuncturist	NS
Minor local bleeding	NS (NS/NS)	Substance-abuse problem	Shenmen, lung, liver, kidney, sympathetic	Unknown number of psychiatrists and nurses	NS

Slight bleeding at insertion	NS (NS/NS)	Heroin addiction	Shenmen, lung, kidney, sympathetic	Unknown number of acupuncturists	NS
Upper limb numbness	NS (NS/NS)	Cholecystolithiasis	Liver, gallbladder, stomach, duodenum, shenmen, sympathetic, lung	Unknown number of TCM practitioners	NS
Pressure ulcers in the pinna	18 (NS/NS)	Chronic nonspecific spinal pain	Shenmen, thalamus	18 doctors and nurses with AT training	NS
Worsened symptoms	8 (NS/NS)	Chronic nonspecific spinal pain	Shenmen, thalamus	8 doctors and nurses with AT training	NS
Mild to moderate local discomfort	5 (NS/NS)	Smoking cessation	Shenmen, lung, mouth, extra, liver	5 acupuncturists	NS
Slight headache	1 (NS/NS)	Smoking cessation	Shenmen, lung, mouth, extra, liver	1 acupuncturist	NS
Sensation of auricular tenderness	2 (NS/NS)	Women with postmenopausal insomnia	Shenmen, kidney, heart, brainstem, subcortex	2 acupuncturists	NS
Mild to moderate local and short-term discomfort	30 (NS/NS)	Persistent allergic rhinitis	Shenmen, internal nose, lung, wind stream	30 acupuncturists	NS
Sore ear	9 (NS/NS)	Persistent allergic rhinitis	Shenmen, internal nose, lung, wind stream	9 acupuncturists	NS
Ear itch	7 (NS/NS)	Persistent allergic rhinitis	Shenmen, internal nose, lung, wind stream	7 acupuncturists	NS
Auricle discomfort without redness or swelling	1 (NS/NS)	Smoking cessation	Lung, shenmen, nicotine, point zero, palate	1 registered nurse	NS

McCulloch, 2015	Cecal intradural hematoma	1 (75/F)	NS	NS	NS	NS
	Bleeding from rupture of extensor tendons by acupuncture needle	1 (81/F)	NS	NS	NS	NS
Wu, 2015	Peripheral nerve injury	8 (26-76, 1NS/4M, 3F, 1NS)	Facial paralysis, left eye pain, cervical dislocation, lateral rectus paralysis, tinnitus and hearing loss, trigeminal nerve pain, lumbago	SJ17, Bitong point, EX-HN07, ST1, ST2, BL2, LI20, SJ21, SI19, around the mandible, GB30, GB34, 1 NS	3 acupuncturists, 1 acupuncturist in the stomatological hospital, 1 private practitioner, 3 NS	1 minute to 3 months
	Cardiac tamponade	4 (19-44/2M, 2F)	Schizophrenia, chronic esophagitis, chest tightness, asthma, diabetes	RN15, chest and back	NS	NS, 3 deaths
	Aortoclasia	2 (42, 64/1M, 1F)	Chest stiffness and rib pain, witchcraft	LR14, limbs, chest, abdomen, and back	NS	NS, 2 deaths
	Cardiac trauma	1 (35/F)	Intercostal neuralgia	Right breast	NS	NS, 1 death
	Chylothorax	1 (21/M)	Bad cold	BL13	NS	2 weeks
	Gallbladder perforation and biliary peritonitis	3 (31-59/1M, 2F)	Stomache ache, cervical spondylopathy, paralytic ileus	ST25, LR14, RN12, RN13, ST25, upper abdomen	NS	NS
	Gallbladder perforation	1 (45/F)	Acute attack of chronic cholecystitis	ST21	NS	NS
	Intestinal perforation and suppurative peritonitis	1 (42/M)	Intestinal spasm	RN8, RN12, ST25	NS	14 days
	Localized peritonitis	4 (37-53/1M, 3F)	Paroxysmal abdominal pain, right lower abdominal pain, abdominal pain, diarrhea, periumbilical pain	Abdomen	NS	NS

Gastric perforation	1 (20/M)	Abdominal discomfort	RN12	NS	11 days
Gastric ulcer with perforation	1 (54/M)	Gastric ulcer	ST36	NS	10 days
Gastric stress ulcer and hemorrhagic shock	1 (61/M)	Rheumatoid arthritis	ST34, ST40, SP6, SP10, GB33, GB34	NS	2 months
Complete intestinal obstruction	1 (2/M)	Diarrhea	RN12, ST25, DU1	NS	NS
Multiple organ injury	1 (45/F)	Chronic bronchitis, coronary disease	GB21, BL13, BL23	NS	NS, 1 death
Cervical common carotid aneurysm	1 (33/F)	Sore throat	Neck	NS	3 months
Sinus caroticus syndrome, shock	1 (42/F)	Thyroid cancer (anaesthesia, for thyroidectomy)	SJ17	NS	NS
Pleural shock	1 (39/M)	Asthma	BL13	NS	2 days
Asphyxia	1 (56/M)	Chest distress	RN22	1 acupuncturist	NS, 1 death
Dyspnea	1 (73/M)	Cough	LI18	NS	3 days
Retinal detachment	1 (53/M)	Blepharospasm	The lateral upper eyelid	1 health worker	NS
Traumatic cataract	1 (63/F)	Left eye ptosis	Periocular	1 acupuncturist	NS
The orbicularis oculi muscle tremor	1 (20/M)	Traumatic mydriasis	EX-HN5	NS	3 days
Speech and swallowing difficulties	1 (35/F)	Right migraine	EX-HN5	NS	2 hours
Femoral neck fracture	1 (40/M)	Schizophrenia	EX-HN5	1 acupuncturist	NS
Flexor halluces muscle and digitorum longus	1 (23/M)	Cramp	BL57	1 health worker	NS

muscle scar contracture					
Nape spasm	1 (17/F)	Shortsightedness	GB20, EX-HN17	1 acupuncturist	6 days
Subluxation of wrist	1 (65/F)	Stroke	LI4, SJ5, LI15	1 intern	1 week
Elbow pain	1 (52/M)	Facioplegia	LI11	1 acupuncturist	2 weeks
Shoulder pain	1 (61/F)	Scapulohumeral periarthritis	NS	1 acupuncturist	20 days
Amyotrophy	1 (53/M)	Cough, epistaxis	LU6	1 private practitioner	NS
Fasciculation	2 (42, 63/2M)	Amyotrophic, lateral sclerosis	Upper limb	NS	NS
Infection	1 (5/F)	Heat, cough	EX-UE19	NS	NS
Abdominal metastatic hepatic hydatid	1 (38/F)	Right upper abdominal mass and discomfort	Ashi points	1 local hospital	NS
Third-degree burns with infection	1 (54/M)	Right leg pain	Local points	1 clinic	NS
Right cheek hematoma with infection	1 (37/F)	Facioplegia	ST5	NS	1 week
Encephalopyosis	1 (30/M)	Insomnia	Head	NS	NS
Pyogenic arthritis	1 (38/M)	Rheumatic arthritis	EX-LE4, EX-LE5, GB34, SP9	1 individual clinic in the country	2 weeks
Diabetes foot	1 (60/F)	Scapulohumeral periarthritis	ST38	NS	3 days
Eye hematoma	1 (28/F)	Chronic conjunctivitis	BL1	1 acupuncturist	14 days
Extraperitoneal hematoma	1 (62/F)	Teratoma of ovary	Hypogastrium	1 roving doctor	NS
Thyroid intracapsular	1 (47/M)	Neck mass	Neck	NS	10 days

hemorrhage with apnea					
Sublingual hematoma	1 (56/M)	Stroke	RN23	NS	1 week
Hematoma compression tracheal cause apnea	1 (50/M)	Cough	ST9	1 unauthorized acupuncturist	NS, 1 dead
Sublingual hematoma	1 (72/M)	Cerebral infarction	EX-HN20	NS	2 days
Orbital hemorrhage	1 (46/F)	Cold headache	EX-HN05	NS	1 month
Hand hematoma	1 (68/M)	Hypertension, stroke	LI4	1 acupuncturist	7 days
Buttock hematoma	1 (65/F)	Neck, waist, and leg pain	Hip	NS	NS
Lower extremity hematoma	1 (61/M)	Cerebral thrombosis	SP6	1 acupuncturist	1 day
Bent	1 (45/M)	Flaccid paralysis	ST36, LI11	1 acupuncturist	NS
Hemopneumothorax	1 (39/M)	Scapulohumeral periarthritis	Supraclavicular fossa	1 country doctor	NS
Sticking of needle	1 (54/M)	The left upper limb dysfunction	Upper limb	NS	NS
Damage of arteria coronaria and cardiac tamponade caused by embedded needle	1 (58/M)	Chronic bronchitis and emphysema	LU1	1 self	NS
Palpitation and paroxysmal pricking pain caused by broken needle	1 (43/F)	Gastric disease	Xiphoid	NS	NS
Pulmonary bulla caused by embedded needle	1 (45/F)	Multiple injuries by traffic	RN23	NS	NS
Broken needle	1 (55/M)	Lumbago	Lower limb	NS	NS

Aphonia	2 (36, 46/2F)	Intercostal neuralgia, obstinate hiccup	PC6	NS	3 days
Hoarseness	1 (43/F)	Neck pain	EX-B05	1 acupuncturist	10 minutes
Fever	1 (52/M)	Cerebral concussion	DU20, GB20, GB30, GB39, LI4, LI11, ST36	1 acupuncturist	NS
Cough	1 (65/F)	Facial neuritis	GB14, BL2, ST2, SI18, RN24, LI4, ST36, LR3	1 acupuncturist	2 minutes
Thirsty	1 (46/F)	Obesity	ST25, ST36, SP15, RN6, LI11, SJ6, SP9, ST40	1 acupuncturist	NS
Infusion reaction	1 (45/F)	Rheumatoid arthritis	Local points	1 acupuncturist	2 hours
Hyperventilation syndrome	1 (35/F)	Cervical pain	GB20, EX-B2	1 acupuncturist	15 minutes
Aggravation of fatigue	1 (46/F)	Nasopharyngeal carcinoma radiation sequela with fatigue	LI11, LI4, ST36, SP6, K13, LU7, SI6, SJ3	1 acupuncturist	NS

Keys: F, female; NS, Not Stated; HIV, human immunodeficiency virus; LHRH, luteinizing hormone-releasing hormone; M, male; MD, medical doctor; NTM, nontuberculous mycobacterial skin infection; OMD, Doctor of Oriental Medicine; PTSD, Post-traumatic stress disorder; TCM, traditional Chinese medicine; TMD, temporomandibular joint disorder; ~, approximately.
